# Global certification of visual impairment registries: A scoping review

**DOI:** 10.1111/aos.16763

**Published:** 2024-09-28

**Authors:** Laura N. Cushley, Benedict Leonard‐Hawkhead, Andrew Jonathan Jackson, Tunde Peto

**Affiliations:** ^1^ Centre for Public Health Queen's University Belfast Belfast UK; ^2^ Department of Ophthalmology Belfast Health and Social Care Trust Belfast UK

**Keywords:** certification, global, partial sight, scoping review, sight impairment, visual impairment

## Abstract

**Background:**

Visual impairment is a global problem which is predicted to rise in the coming years. Some of the biggest causes of visual impairment globally include uncorrected refractive error, cataract and age‐related macular degeneration. People with a visual impairment often require support and so many countries hold registers of visual impairment. These registers can sit at a national, regional or local level. This scoping review aims to identify which countries hold visual impairment registries and have published data from them.

**Methods:**

Medline All, Embase and EBSCOHost were searched using several search terms after consulting an information specialist. All papers after the year 2000 were included in the scoping review. All results are shown using a PRISMA diagram and presented narratively.

**Results:**

The total number of articles and papers identified was 1266; after screening and review, 57 articles were included in the review from 2000 to 2024. These articles came from 19 different countries and encompassed national, regional and local visual impairment databases. Many countries cited age‐related macular degeneration as the major cause of blindness with diabetic retinopathy and glaucoma following. In less economically developed countries, refractive error was the main cause of sight loss. There were papers which focused on specific eye conditions such as glaucoma and diabetic retinopathy or on specific cohorts including working‐age population and children. The leading causes of blindness in children appeared to be inherited retinal diseases, albinism and cerebral visual impairment.

**Conclusion:**

Certification of visual impairment is held differently across the world. There is commonality among different countries regarding the major causes of visual impairment in both adults and children. The importance of holding visual impairment registers to support people with a visual impairment and to plan services is essential.

## 1 INTRODUCTION

Visual impairment affects 2.2 billion people worldwide with a predicted rise of as much as 55 percent in the next 30 years (International Association for the Prevention of Blindness, 2020). The WHO defines mild vision impairment as anything below 6/12 (0.3 LogMAR), moderate as anything worse than 6/18 (0.477 LogMAR), severe vision impairment as worse than 6/60 (1.0 LogMAR) and blindness as below 3/60 (1.3 LogMAR) in the better eye (World Health Organization, 2019). Reports suggest that globally in 2015, it was estimated that 36 million people were blind, 217 had a moderate to severe visual impairment and 188 million had a mild vision impairment (Bourne et al., 2021).

Globally, the most common causes of visual impairment are uncorrected refractive error, cataract, diabetic retinopathy, glaucoma and age‐related macular degeneration but there is a large variation in most common causes between countries and continents (World Health Organization, 2023). Due to the aging population worldwide, there will be an additional burden of visual impairment accompanying the rise in multi‐morbidities, such as the rise of diabetes mellitus and its associated ocular complications, including early cataracts (International Diabetes Federation, 2019, 2020). Living with visual impairment can result in challenges with daily life tasks (Fenwick et al., 2012; Lamoreux et al., 2008; Nutheti et al., 2006; Sharma et al., 2005, 2014; Tyler, 2011; Woodcock et al., 2004), added financial burden (Pezzullo et al., 2018), social isolation (Alliance for Aging Research Team, 1999; Gallagher et al., 2011) and other physical (Hong et al., 2014) and mental health problems (Nollett et al., 2016; Slade & Edwards, 2015).

In order to provide support for people with visual impairment, many countries established registers to serve this purpose. Registers provide the relevant governmental and non‐governmental organisations, sensory service teams and social workers with a list of people known to have visual impairment. In most countries, registration/certification of visual impairment is voluntary and benefits might include financial help, protection under equality acts and access to get practical help with vision loss. These data also contribute to building a knowledge base for understanding visual impairment across a region/country for planning future services. There have been several studies published on data from the UK including from England & Wales, Scotland and Northern Ireland (Bamashmus et al., 2004; Bunce et al., 2010, 2015, 2017; Bunce & Wormald, 2006, 2008; Durnian et al., 2010; Jackson et al., 2020, 2021; Liew et al., 2014; Malik et al., 2012; Mitry et al., 2013; Quartilho et al., 2016; Rahman et al., 2020; Rees et al., 2014; Savage et al., 2018; Thomas et al., 2017). However, our understanding on a global scale is still scant.

This scoping review was designed to determine the process of certification of visual impairment globally, how data are being collected in different countries/regions and what are the major causes of visual impairment globally. Therefore, papers involved in this scoping review are only included if they are using a sight impairment certification/registration database.

## 2 METHODS

An information specialist (RF) was consulted on database search and search terms. Three databases were searched on 25/07/2023 – Medline All (Shibboleth), Embase (Shibboleth) and EBSCOHost (CINAHL Complete). The search terms used for all databases were certification OR registration OR register AND partial blindness OR blindness OR visual impairment OR vision loss OR sight loss OR sight impairment OR low vision. Only papers published after 2000 were included to keep the review relevant.

All papers were imported into Rayaan AI software for review. All abstracts were screened independently by two reviewers for inclusion and exclusion for review. No artificial intelligence was used to screen papers in Rayyan AI – all papers were manually reviewed by the two reviewers in parallel. Rayann AI was solely used to reject/include papers and ensure blind review between reviewers and flag conflicts. Any conflicts were discussed by the two reviewers and if a decision was not reached then a third, independent reviewer was consulted. Full‐text papers and articles for all eligible abstracts were then downloaded from the appropriate journals and uploaded into Rayann for independent review by both reviewers. Reasons for rejecting most studies were that they were randomised controlled trials (many of which were not ophthalmology), systematic reviews and studies not using a sight impairment register.

Once included, studies were finalised and one reviewer extracted all necessary data from the papers. These data included country of registrations, eye conditions, year of data, who collected the data, whether it is a formal or informal database and geographic area of database coverage. A secondary re‐run of the search was completed on 13/08/2024 for years 2023–2024 to update the scoping review. All three databases were searched using the same terms as described above. All abstracts and titles were reviewed by the same two reviewers in parallel and followed the above methodology.

## 3 RESULTS

**FIGURE 1:**
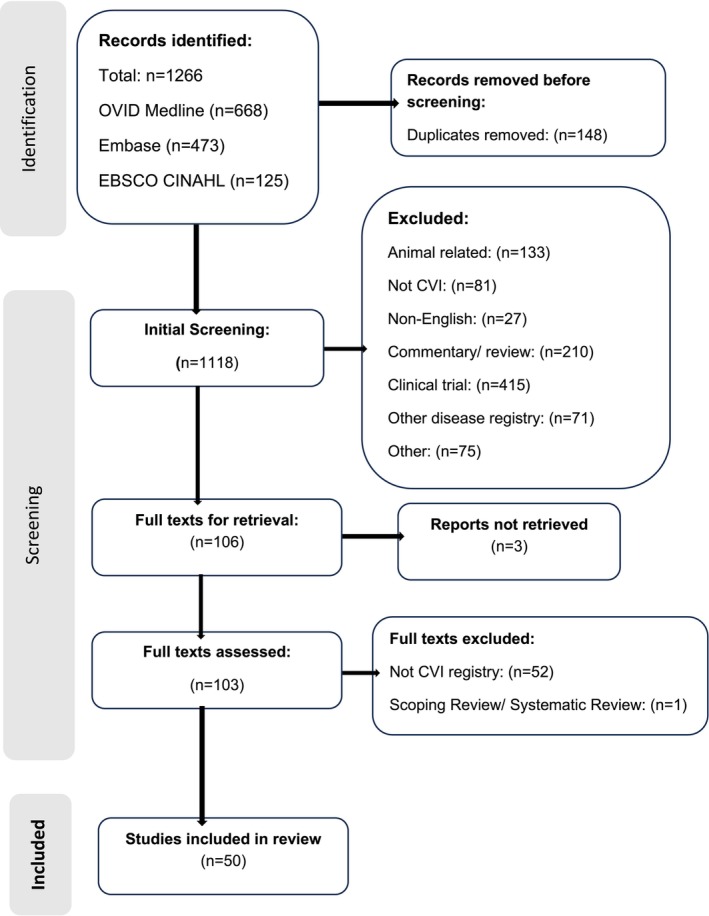
Preferred reporting items for systematic reviews and meta‐analyses (PRISMA) flowchart of global certification of visual impairment (initial search).

In total, 50 articles were included in the review and spanned from 2000 to 2023. Figure 1 below shows the PRISMA (Page et al., 2021) diagram demonstrating the screening process. In the additional re‐run of the search terms, a further 116 papers were identified. Twenty‐ eight papers were removed as they appeared in the initial literature search, which partly included the year 2023. Two were removed as they were duplicated across the databases. On review, there were 18 conflicts and after conflict resolution, 14 papers were identified for full text review. From those, 1 was removed as it was a device register, 1 was removed as it was a survey not registry, 2 were removed as they were studies not using registries, 1 was removed as it was a comment editorial and 1 was removed as it was not a sight registry. There was an addition of seven papers in the scoping review. It should be noted that Figure 1 depicts the initial screening of papers and does not reflect the updated literature search noted above.

### 3.1 Study characteristics

Publications on the topic originated from 19 different countries, mostly from high‐income countries (Table 1). Forty‐five (78.9%) studies included data from a national/countrywide registry, seven contained data collected within a clinic/hospital, two were state or district registries, one was a charity registry, one was a school registry, one was an original population‐based cohort and one was a disease‐specific registry on ROP. Data were collected between 1 and 40 years. Altogether 23 studies reported on all eye conditions – ten were on childhood blindness, another nine were on diabetic retinopathy, five were on AMD, two on inherited retinal conditions and two on glaucoma. One study reported on adolescents, Asian and Caucasian causes, uveitis, bilateral blindness and working‐age adults.

**TABLE 1:** Table of included study countries and eye conditions reported on.

Country	Eye conditions	Country	Eye condition reported
Australia	All diseases, AMD, IRD, Childhood blindness, CVI	Northern Ireland	All diseases
China	All Diseases, Vitreoretinal diseases	Norway	Childhood blindness
Croatia	IRDs	Oman	Bilateral blindness
Denmark	AMD, Pre‐term births, Childhood blindness	Poland	DED
England	All Diseases (with Wales), AMD, DED, Glaucoma, Uveitis, Childhood blindness	Republic of Ireland	All diseases
Finland	AMD, DED, Glaucoma, Childhood blindness	Scotland	All diseases
India	All diseases	Sweden	Childhood blindness, ROP
Israel	All diseases, Childhood blindness	Taiwan	All diseases
Japan	All diseases	Trinidad	All diseases
		Wales	All diseases (with England), DED

Abbreviations: AMD, age‐related macular degeneration; CVI, cerebral visual impairment; DED, diabetic eye disease; IRD, inherited retinal diseases; ROP, retinopathy of prematurity.

Finland, Oman, Israel, Croatia, Republic of Ireland and Taiwan were reported to have national databases. England & Wales, Northern Ireland and Scotland have regional databases. Australia and Poland have state/province‐wide databases. Sweden had no national registry, and the Norway national registry ended in 1995. Japan has a nationwide database of welfare offices. Trinidad's results were cross‐validated among two different registries. China has a register of vitreoretinal disease (Table 2).

**TABLE 2:** Table of reported most common causes of visual impairment in adults and children according to studies including all diseases.

Main cause of visual impairment	Country
Adults	
Age‐related macular degeneration (AMD)	England, Israel, Wales, Australia, Republic of Ireland, Scotland
Myopia	China
Glaucoma	Trinidad, Japan, Taiwan
Children	
Optic nerve abnormalities	Israel, Finland
Inherited retinal diseases (IRDs)	Australia
Cerebral visual impairment (CVI)	England, Denmark
Neuro‐ophthalmic disorders	Sweden

### 3.2 All eye diseases

Many studies showed that AMD was the commonest cause of visual impairment in databases originating from England and Wales, Israel, Northern Ireland, Republic of Ireland, Norway, Scotland and Australia (Bamashmus et al., 2004; Bloch et al., 2012; Bunce et al., 2010; Bunce & Wormald, 2006, 2008; Israeli et al., 2022; Kelliher et al., 2006; Malik et al., 2012; Quartilho et al., 2016; Savage et al., 2018; Skaat et al., 2012; Yong et al., 2006). In many of these countries, certification and registration rates due to AMD are around 50% of total certifications. In contrast, a local audit in Royal Cornwall showed that they had lower rates of AMD compared to regional data (Savage et al., 2018).

In Japan, China and Taiwan, AMD was not the leading cause of blindness and instead was replaced by retinal diseases, glaucoma and myopic macular degeneration (Kareemsab et al., 2011; Matoba et al., 2023; Tsai et al., 2008; Wu et al., 2011). In Trinidad, the leading cause was glaucoma alongside retinal detachment, trauma, cataract, AMD and retinal dystrophy with the biggest cause being glaucoma (Ramsewak et al., 2024). In India, the leading causes of blindness were congenital abnormalities (22%), refractive error (19%) and retinitis pigmentosa (18%), AMD and glaucoma only accounted for 5% each of all causes (Kareemsab et al., 2011).

### 3.3 Studies on AMD

Studies on AMD were all from more economically developed countries with national registers. In these studies, England and Finland showed that more females than males were certified with AMD (Purola et al., 2023; Rees et al., 2014). Studies from England and Finland also showed the percentage of people registered annually with geographic atrophy (GA) fell between 40–50% with 28–35% having neovascular AMD (Bunce et al., 2015; Purola et al., 2023; Rees et al., 2014). All studies showed that there was a decrease in the prevalence of AMD‐related visual impairment after the introduction of anti‐VEGF injections (Bloch et al., 2012; Bunce et al., 2015; Jeffery, Mukhtar, Lopez, et al., 2021; Purola et al., 2023; Rees et al., 2014).

### 3.4 Studies on diabetic retinopathy and diabetic eye disease

There were eight studies on diabetic retinopathy (DR) which showed contrasting results. In Finland and Poland, there was an overall/significant reduction in registrations with diabetic eye disease (Bandurska‐Stankiewicz & Wiatr, 2006; Heloterä et al., 2024; Purola et al., 2022). In North and East Devon, England, there was no significant increase or decrease in registrations due to DR (Lin et al., 2017) and in Wales, there was a prevalence increase in DR (Thomas et al., 2017). Finland also showed that people were being registered with a visual impairment due to DR older and with a decreased severity (Laatikainen et al., 2016). A study in England showed that men had a lower risk of visual impairment and people in more deprived areas were at higher risk for visual impairment (Olvera‐Barrios et al., 2023).

### 3.5 Glaucoma

A study in Trinidad showed that glaucoma was the biggest cause of visual impairment in the country (Ramsewak et al., 2024). Finland showed that overall, the incidence of reported visual impairment due to glaucoma had increased; however, the incidence of visual impairment in the glaucomatous population had decreased (Vaajanen et al., 2022). This study also showed that the incidence had increased in women over the age of 40 (Vaajanen et al., 2022). Fife in Scotland also showed that there was an increase in certifications due to glaucoma. Interestingly, the same study showed that over one‐third of those certified with glaucoma in Fife had a cognitive impairment (O'Colmain et al., 2011).

### 3.6 Inherited retinal degenerations

Studies in Australia showed that inherited retinal degeneration was the second most common cause of visual impairment in adults after AMD (Jeffery, Mukhtar, Mcallister, et al., 2021). Similarly, it is the second biggest cause of visual impairment in children and the most common in working‐age people (Jeffery, Mukhtar, Mcallister, et al., 2021). Croatia shows a similar story with age‐related causes being the biggest cause, followed by IRDs (Kukulj & Zoric‐Geber, 2002). The Croatian study also showed there was a male dominance in IRD certifications (Kukulj & Zoric‐Geber, 2002). England and Wales further reflect this with IRD now the leading cause of visual impairment in working‐age people, taking over from DR in 1999–2000 (Liew et al., 2014). In Israel, IRDs were found to be a common cause of visual impairment in the working‐age population; however, optic atrophy was found to be the leading cause (Merrick et al., 2004).

### 3.7 Childhood blindness

Data from England indicate that there was an increase in the incidence of childhood blindness overall (Bunce et al., 2017; Mitry et al., 2013) whereas in Israel, childhood blindness rates have remained stable (Israeli et al., 2023). A study in Denmark showed that fewer children are severely visually impaired at the time of registration now than in previous years (Kessel et al., 2024) and blindness has been decreasing in pre‐term children over the last four decades(Al‐Abaiji et al., 2024). Many countries show similar causes of registration such as IRDs, cerebral visual impairment (CVI) and albinism (Haugen et al., 2016; Mitry et al., 2013; Rudanko & Laatikainen, 2004; Silveira et al., 2022). In Denmark, the biggest causes of childhood blindness were CVI and optic nerve atrophy (Kessel et al., 2024) and in pre‐term children, they were retinopathy of prematurity (ROP), optic atrophy and CVI. A Swedish study showed that 14% of children with ROP have a visual impairment (Larsson et al., 2024). In Finland, ocular malformation and neuro‐ophthalmic disorders were common causes of registration (Rudanko & Laatikainen, 2004) and in Norway, neuro‐ophthalmic disorders were the most common cause of registration (Haugen et al., 2016). Many studies showed a link between childhood visual impairment and co‐morbidities. In Australia, 44% of children were known to have co‐morbidity (Silveira et al., 2022) and they have completed a study specifically on cerebral visual impairment (CVI) (Silveira et al., 2023). Both in Norway and in Sweden, over half of the children (53% and 55%, respectively) had additional functional impairments (motor or functional; Blohmé et al., 2000; Haugen et al., 2016).

## 4 DISCUSSION

Altogether over 1000 papers were identified through initial screening, only 50 studies were finally included in this scoping review. The most common reason for removing papers was due to the search term ‘blind’ being used, as this brought in many clinical trials using allocation concealment and thus was not relevant to our research question. In addition, many studies reported on registers that included visual impairment data, such as cerebral palsy registers, but were not visual impairment registers themselves.

Studies included were from countries on the continents of Asia, Australia and Europe with a notable lack of registers published from North/South America or Africa. This does not necessarily mean the lack of registers of visual impairment; it might just mean that such publications are not easily recognisable by search engines. Other reasons include an ad‐hoc nature of collecting data, especially in lower economic settings and vast sizes of the countries and different states/territories. Of the studies included, many were from the same database on different age groups (such as children and working age) and causes (AMD, DR and glaucoma). Data from the United Kingdom alone accounted for 21 studies. In many high‐income countries, the most common cause of visual impairment was AMD, consistent with the aging population. While AMD remains the biggest cause of visual impairment in many countries, accounting for approximately 50% of total registration, studies have shown that registration had decreased after the introduction of anti‐VEGF treatment (Bloch et al., 2012; Bunce et al., 2015; Jeffery, Mukhtar, Lopez, et al., 2021; Purola et al., 2023; Rees et al., 2014). To reflect this, the International Association for the Prevention of Blindness (IAPB) World Atlas shows that AMD is the third largest cause of vision impairment after refractive error and cataract (International Association for the Prevention of Blindness, 2020). In contrast however, on the Asian continent, AMD only contributed around 7% of the registrations (Flaxman et al., 2017; Kareemsab et al., 2011; Matoba et al., 2023; Tsai et al., 2008; Wu et al., 2011), with myopic macular degeneration being in the first position followed by retinal diseases and glaucoma (Kareemsab et al., 2011; Matoba et al., 2023; Tsai et al., 2008; Wu et al., 2011). In India, uncorrected refractive error was the most common cause, and this is consistent with the lack of infrastructure for refractive spectacle availability (Kareemsab et al., 2011).

Studies in Finland and Poland on diabetic retinopathy generally showed that there had been a significant reduction in registrations and in England and Wales, there was no recent change. This is likely due to the implementation of diabetic eye screening services in multiple countries including the UK since the 2000s. In addition, evidence from Finland showed that although people are being diagnosed with having DR and some become visually impaired when they do, they are older and the severity of impairment is less (Laatikainen et al., 2016). This is in contrast with the fact that diabetic retinopathy is a growing cause of vision loss globally (International Association for the Prevention of Blindness, 2020). This is likely due to the lack of screening and treatment in many countries including lower economically developed countries.

Glaucoma is one of the leading causes of visual impairment in many countries including the UK (Bunce & Wormald, 2006) and in some countries, the numbers are increasing (Laitinen et al., 2010). It was interesting to see that while numbers of people registered annually with glaucoma have increased, the incidence of the glaucomatous population has decreased. Due to the aging population, the number of people affected with glaucoma is increasing, therefore it is important to monitor the incidence of visual impairment and its severity. Interestingly, the study in Fife, Scotland showed that many with glaucoma‐related registration also had a cognitive impairment as well (O'Colmain et al., 2011). This could potentially affect glaucoma treatment adherence as well.

Studies suggest that there is a disease burden shift from communicable to non‐communicable diseases globally. AMD, diabetic retinopathy, glaucoma and cataract are the diseases often associated with this shift (Bourne et al., 2021). One study in mainland China included within the scoping review found that women, older people and those from Midwest China were more likely to suffer vision loss. This is in keeping with many studies linking vision impairment and blindness to reduced economic, educational and employment opportunities (Eckert et al., 2015; Frick et al., 2015; Frick & Foster, 2003; Reddy et al., 2018). The IAPB also states that vision loss is driven by inequality (International Association for the Prevention of Blindness, 2020). This is outside the scope of this review. In addition, studies suggest that age plays a factor (Flaxman et al., 2017) but many of the studies in this review do not focus on this or give an age range therefore it is outside the scope.

Interestingly, IRDs were found to be the second most common cause of visual impairment in Australia in adults, after AMD, including in the working‐age population (Jeffery, Mukhtar, Mcallister, et al., 2021). This would be in contrast with UK data and certainly data in Northern Ireland (Jackson et al., 2020) where glaucoma and diabetic eye disease are often listed as the most common causes after AMD. In Israel, IRDs are a common cause of visual impairment – however, optic atrophy is the leading cause. As diabetes‐related eye diseases are no longer the leading cause of visual impairment in the working‐age population in England, Wales and Northern Ireland, IRDs may be showing a proportional rise in those countries where treatable diseases are properly taken care of (Cushley et al., 2023; Liew et al., 2014).

Included studies on childhood visual impairment showed that while there was no change in the incidence of visual impairment in Israel (Israeli et al., 2023), in England, there was an increase in numbers registered (Bunce et al., 2017; Mitry et al., 2013) and in Demark, the numbers reduced (Al‐Abaiji et al., 2024; Kessel et al., 2024). This could be attributed to concerted efforts to make more people aware of registration and its benefits. It could also be due to more knowledge and awareness in the community in relation to children's eye conditions including cerebral visual impairment and IRDs. The nomenclature has changed somewhat and there are alternative ways of referring to conditions such as cerebral visual impairment including cortical visual impairment or neuro‐ophthalmic disorder. There is also a more detailed understanding of multiple co‐morbidities, often accompanying other additional and complex needs such as in cerebral palsy (Philip et al., 2020; Schenk‐Rootlieb et al., 1994) and Down syndrome (Krinsky‐McHale et al., 2014). This is further supported by the studies which show an average of between 40 and 50% of certifications in children having additional needs listed (Blohmé et al., 2000; Haugen et al., 2016; Silveira et al., 2022).

## 5 CONCLUSIONS

Certification of visual impairment seems to show a wide range of how it is administered in different countries ranging from national registers, regional and territory‐specific registers. This scoping review revealed that 18 countries published outcomes of their visual impairment registers and that one, in Norway, was no longer in place. The different patterns on different continents show that targeted interventions are likely to be needed if we are to reduce the overall impact of visual impairment. Diagnostic and referral criteria might also differ between populations. Access to services and benefits might change the population's willingness to be registered, especially if they might even perceive that they will be discriminated against if they have a known disability. The studies included show how keeping a register of visual impairment might add to our understanding of service provision to such a population.

## AUTHOR CONTRIBUTIONS

LNC, AJJ and TP drafted the research protocol; LNC drafted the search terms and consulted an information specialist; LNC and BLH completed the initial and secondary screening of the articles; LNC completed the data analysis; LNC drafted the manuscript; BLH, AJJ and TP reviewed and edited the manuscript; LNC corrected and edited the final manuscript for submission; and LNC involved in the submission and correspondence of the manuscript.

## FUNDING INFORMATION

This project was supported by funding from Charitable Funds at the Belfast Health and Social Care Trust and the Belfast Association for the Blind.

## CONFLICT OF INTEREST STATEMENT

No conflicts of interest to declare.

## Data Availability

The dataset/articles supporting the conclusions of this article are included within the article.
